# Enpp1 mutations promote upregulation of hedgehog signaling in heterotopic ossification with aging

**DOI:** 10.1007/s00774-024-01543-1

**Published:** 2024-08-30

**Authors:** Zhongyuan He, Zhengya Zhu, Tao Tang, Fuan Wang, Peng Guo, Jianfeng Li, Nguyen Tran Canh Tung, Qian Liang, Shaoyu Liu, ManMan Gao, Xizhe Liu, Zhiyu Zhou

**Affiliations:** 1 Innovation Platform of Regeneration and Repair of Spinal Cord and Nerve Injury, Department of Orthopaedic Surgery, The Seventh Affiliated Hospital, Sun Yat-sen University, Shenzhen, 517108 China; 2 Department of Orthopaedics, The Second Affiliated Hospital of Chongqing Medical University, Chongqing, 400016 China; 3 Guangdong Provincial Key Laboratory of Orthopedics and Traumatology, The First Affiliated Hospital of Sun Yat-sen University, Guangzhou, 510062 China; 4 Department of Sport Medicine, Inst Translat Med, The First Affiliated Hospital of Shenzhen University, Shenzhen Second People’s Hospital, Shenzhen, 518025 China; 5 Department of Orthopaedic Surgery, Faculty of Medicine, University of Toyama, Toyama, 930-0194 Japan; 6 Department of Spine Surgery, The First Affiliated Hospital of Shenzhen University, Shenzhen Second People’s Hospital, Shenzhen, 518025 China

**Keywords:** Enpp1, HOTL, OPLL, Hedgehog signaling

## Abstract

**Introduction:**

Heterotopic ossification of the tendon and ligament (HOTL) is a chronic progressive disease that is usually accompanied by thickening and ossification of ligaments and high osteogenic activity of the surrounding ligament tissue. However, the molecular mechanism of maintaining the cellular phenotype of HOTL remains unclear.

**Materials and methods:**

We first constructed a model of HOTL, *Enpp1*^*flox/flox*^*/EIIa-Cre* mice, a novel genetic mouse system. Imaging, histological, and cell-level analyses were performed to investigate the progressive ossification of the posterior longitudinal ligament, Achilles tendons, and degeneration joints caused by Enpp1 deficiency.

**Results:**

The results indicate that Enpp1 deficiency led to markedly progressive heterotopic ossification (HO), especially spine, and Achilles tendons, and was associated with progressive degeneration of the knees. The bone mass was decreased in the long bone. Furthermore, fibroblasts from *Enpp1*^*flox/flox*^*/EIIa-Cre* mice had greater osteogenic differentiation potential following induction by osteogenesis, accompanied by enhanced hedgehog (Hh) signaling. In addition, fibroblast cells show senescence, and aggravation of the senescence phenotype by further osteogenic induction.

**Conclusion:**

Our study indicated that with increasing age, mutations in Enpp1 promote ectopic ossification of spinal ligaments and endochondral ossification in tendons and further aggravate knee degeneration by upregulating hedgehog signaling.

**Supplementary Information:**

The online version contains supplementary material available at 10.1007/s00774-024-01543-1.

## Introduction

The heterotopic ossification of tendons and ligaments (HOTL) is a common degenerative disease in clinics that exhibits identical tissue characteristics, pathology, and mechanisms. Unfortunately, the lack of understanding of cellular and molecular mechanisms has prevented the development of effective treatment [1–5]. One specific type of HOTL is posterior longitudinal ligament ossification (OPLL), a chronic myelopathy resulting from abnormal ossification of spinal ligaments. OPLL's etiology involves a complex interplay of genetic, intracellular, external, and biomechanical factors [6–8]. Although epidemiologic studies have suggested a genetic predisposition for OPLL, the specific mode of inheritance and mechanism has not been elucidated [9, 10].

To better comprehend OPLL and HOTL, researchers have turned to animal models. Tiptoe walking (ttw) mice were initially identified in the ICR mouse strain and were found to carry a nonsense mutation (Gly568Stop) in the Enpp1 gene. The mice exhibit accelerated bone growth and have become a valuable tool for studying OPLL [11]. Enpp1 is an ectoenzyme that generates inorganic pyrophosphate (PPi) and plays a role in bone mineralization and soft tissue calcification by regulating pyrophosphate levels [12]. Histological and biochemical studies show similarities in spinal ligament abnormalities in patients with ttw mice and patients with OPLL [13–15]. Nonetheless, ttw mice are hard to obtain; adult ttw mice have low fertility, and cannot perform conditional knockout of the Enpp1 gene, so there is a limitation in specifically simulating spine-related ligament ossification.

In humans, Enpp1 defects result in conditions like generalized arterial calcification and pseudoxanthoma elasticum [16–18]. Additionally, recent evidence indicates that both heterozygous and compound heterozygous pathogenic variants in ENPP1, which cause Autosomal Recessive Hypophosphatemic Rickets Type 2 (ARHR2) as well as Generalized Arterial Calcification of Infancy (GACI), may also increase the risk for DISH and OPLL. Excessive production of FGF23 in bones can lead to renal phosphate depletion, hypophosphatemia, and impaired vitamin D metabolism, resulting in inadequate bone mineralization, such as rickets and osteomalacia, ARHR2 may manifest in survivors of GACI [19, 20]. Enpp1 impacts gene expression and vitamin D metabolism, contributing to age-related conditions and ectopic calcification [21]. This includes effects on cartilage, ligaments, and joint health. Enpp1's potential involvement in OPLL becomes more evident, as ligament tissue from OPLL patients shows significantly higher Enpp1 expression than in healthy subjects. [22, 23] Studies in mice lacking Enpp1 function demonstrate ectopic calcification and osteoarthritis-like changes [24, 25]. Manipulating PPi levels using exogenous PPi and an alkaline phosphatase inhibitor shows promise in slowing ossification progression [26]. OPLL has been reported to be associated with endochondral ossification, but the specific mechanism is unknown. The hedgehog (Hh) signaling pathway emerges as a key player in embryonic limb patterning, chondrocyte differentiation, and osteogenesis during long bone growth [27–29]. This pathway's activity influences cell differentiation and is mediated by hedgehog ligands (SHH, IHH, DHH) and GLI transcription factors. Additionally, osteoblast-related GSα gene expression is upregulated, potentially contributing to progressive ossification [30–32]. Therefore, we speculate whether the hedgehog (Hh) signaling pathway plays an important role in OPLL lacking ENPP1 expression. In the study, *Enpp1*^*flox/flox*^*/EIIa-Cre* mice can be used as an effective model to study OPLL. Further, conditional knockout of ENPP1 in time and space can help to study its specific functions. In addition, to regulating PPi homeostasis, ENPP1 deficiency may also promote ossification by activating the Hh signaling pathway, leading to OPLL. Ultimately, these investigations aim to bridge the gap in understanding the cellular and molecular mechanisms underlying HOTL, offering insights into potential treatment strategies.

## Materials and methods

### Animals and diet

**Fig. 1:**
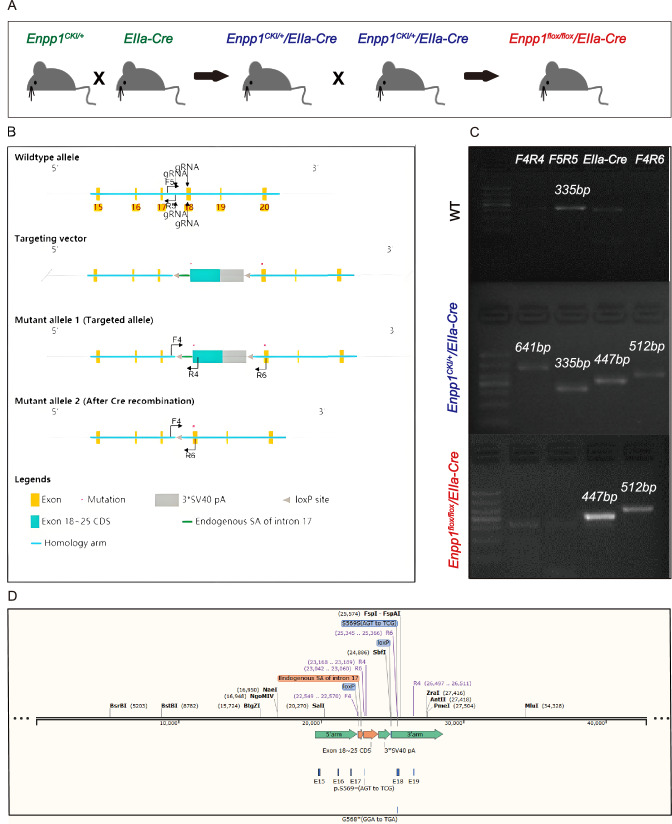
Gene mouse construction process. **A** Mouse hybridization process. **B** The construction process of Enpp1^CKI /+^ mice and *Enpp1*^*flox/flox*^*/EIIa-Cre*, the schematic of gene mouse identification. The p.G568*(GGA to TGA) mutation will be introduced into Exon 18 through gRNA cleavage. **C** Results of gene identification in the tail of WT, Enpp1^CKI/+^/EIIa-Cre, and *Enpp1 *^*flox/flox*^*/EIIa-Cre* mice. **D** Sequencing results of tail DNA sequence of *Enpp1 *^*flox/flox*^*/EIIa-Cre* mice

The construction process of homozygote *Enpp1*^*flox/flox*^*/EIIa-Cre* mice is shown in Fig. 1A. Enpp1^CKI/+^ conditional targeted mice were generated as described in Fig. 1B by Cyagen Biosciences Inc. (Guangzhou, China). The mouse Enpp1 gene (NCBI Reference Sequence: NM_008813.4) is located on mouse chromosome 10. For the KI model, the “loxP-Endogenous SA of intron 17-Exon 18–25 CDS-3*SV40 pA-loxP” will be inserted into intron 17, and the p.G568* (GGA to TGA) mutation will be introduced into Exon 18 through gRNA cleavage. A silent mutation will also be introduced into the “Exon 18–25 CDS” cassette and Exon 18 to prevent the binding and re-cutting of the sequence by gRNA after homology-directed repair. To engineer the targeting vector, homology arms will be generated by PCR using BAC clone RP23-85B3 as a template. Cas9 and gRNA will be co-injected into fertilized eggs using a targeting vector for mice production. A PCR test will be performed on the pups, followed by sequencing analysis. EIIa-Cre tool mice were obtained from Cyagen Biosciences Inc. (Guangzhou, China). EIIa-cre mice carry a cre transgene under the control of the adenovirus EIIa promoter that targets Cre recombinase expression in the early mouse embryo and is useful for germline deletion of loxP-flanked genes.

The genetically modified mice were raised in-house and weaned at 3–4 weeks of age, and the mice were maintained on a normal laboratory diet. All behavioral experiments were performed during the same circadian period, and all mice were maintained on a 12 h:12 h natural light: dark cycle (8:00 a.m to 8:00 p.m). Our experiments were conducted with only male mice because it is necessary to breed the appropriate number of female mice to obtain the desired genotypes for genetic experiments. Genotyping was performed by standard PCR-based procedures using tail genomic DNA. The sample size was estimated based on previous work and published literature. All procedures were in accordance with protocols approved by the Institutional Animal Care and Use Committee, Sun Yat-Sen University.

### Mouse tail genotype identification

Mouse genotyping was performed using standard protocols followed by sequencing analysis. In brief, 2 mm tail fragments were clipped when the mice were 2 weeks old, digested using a test mouse genotyping kit (MG500, Genecopoeia), inactivated, and subjected to DNA amplification. PCR was performed using Pro Taq Master Mix, dye plus (AG11112, Accurate Biology AG, CHN) on a Real-Time System (Bio-Rad). Each reaction mixture was 25 μl and contained 1 μl of the cleaved product, 12.5 μl of 2 × Master Mix, 10.5 μl of nuclease-free water, and 0.5 μl each of 10 μM. The following cycle conditions were applied: 94 °C for 30 s, 55 °C for 30 s and 72 °C for 2 min for 34 cycles; 72 °C for 7 min; and finally kept at 4 °C. The specific primers used in this study were designed using Primer 6.0 software (Applied Biosystems, Foster City, CA, USA), and the sequences of the forward and reverse primers were as follows: F4, 5′-TCAGGAATCTGGGTGGCATAGC-3′, R4, 5′-TTGATTGGCACAATCCAAGGGT-3′; F5, 5′-GACAAGTGGACTTTGGCTTCTGT T-3′, R5, 5′-TTCTCCACCCCAAATGCGCTG-3′; EIIa-M-F2, 5′-TGGCCGCTGGAG ATGAC-GTAGTTT-3′, EIIa-M-R, 5′-GAACATCTTCAGGTTCTGCGGG-3′; and R6, 5′-TTAAGGCTGCCATGCGATCAAT-3′. To further confirm the successful location of the mutation, mouse tail DNA was amplified with the primer F4R6 and subjected to DNA sequencing (Tsingke Biotechnology).

### Assessment of body weight and behavioral function

All animals underwent body weight measurements once a week until 28W of age. Three unbiased observers (ZY.H, ZY.Z, T.T) analyzed neurological and hindlimb performance using the BMS. Three observers performed ROM measurements and anesthetized the mice with isoflurane low-flow inhalation and placed them on a horizontal tabletop. A full extension of the ankle joints of the mice was limited to the horizontal plane, and the hindlimb movement was restricted to the horizontal plane. The ankle ROM was measured using a goniometer, which measures the angle between the tibial long axis and the plantar aspect of the hind foot.

### Micro-CT analyses

The HOTL process was observed using micro-CT analyses performed on mice of different ages (Scanco medical, μCT100, Bassersdorf, Zurich, Switzerland). To detect ectopically mineralized components, the data were analyzed at a threshold of 255. CTvol, CTAn, and DataViewer software was used to analyze the reconstructed images.

### Isolation of ligament fibroblasts from mice

Drawing from prior literature, novel methods were formulated for the acquisition and cultivation of primary ligament fibroblasts [33]. Under a dissection microscope, the PLL of mice was carefully excised from a non-ossified site to avoid the possibility of contamination with osteogenic cells. The ligaments were minced into approximately 1–3 mm pieces after being rinsed with phosphate-buffered saline (PBS) to remove blood and debris. Following ligament fragment dissociation, collagenase I was used to extract cells from ligament fragments (Gibco, USA, 17100017). The cells were harvested every half hour and cultured in Dulbecco’s modified Eagle’s medium (Dmem; Gibco, Grand Island, NY, USA) supplemented with 10% fetal bovine serum and antibiotics at 37 °C in 5% CO_2_.

### In vitro osteogenic induction

The cells at P1 were seeded in 6-well plates at the density recommended previously in the culture medium. The osteogenic medium was applied after the cells had reached 70–80% confluence, consisting of 50 μg/ml ascorbic acid (A8960, Sigma), 10 mm β-minimal medium replacement with glycerophosphate (G9422, Sigma), and 100 nM dexamethasone (D4902). To facilitate osteogenic differentiation, the osteogenic medium was replaced every 2 days for a period of 14 and 28 days [34].

### Histochemistry and immunofluorescence

After the mice were systemically perfused with paraformaldehyde at 4W, 8W, and 28W time points, the spine, knee joints, and Achilles tendons were harvested and washed three times for half an hour each time in PBS at 4 °C. The tissues were decalcified with 12.5% EDTA and embedded in OCT. Frozen sections were sliced to a thickness of 10 mm. Non-decalcified Achilles tendons were used for A For staining, non-decalcified Achilles ten-dons were subjected to Alizarin Red staining, while other stains used decalcified tissues. Cells were fixed and incubated in 1% Alizarin Red S for 10 min, followed by rinsing with distilled water. Images were then captured. Standard protocols were employed for HE, ALP, and Alcian Blue & Nuclear Fast Red Staining, and images were captured using the Leica DM4B system and Digital Pathology Section Scanner (KF-PRO-005, KFBIO technology). Immunofluorescent staining was carried out by incubating samples overnight at 4 °C with antibodies such as OCN, OPN, IBSP, RUNX2, COL2A1, SOX9, SHH, PTCH, GLI1, SUFU, and Vimentin. Subsequently, the samples were incubated with secondary antibodies, donkey anti-mouse IgG (H + L) Highly Cross-Adsorbed Secondary Antibody, Alexa Fluor Plus 594, and donkey anti-rabbit IgG (H + L) Highly Cross-Adsorbed Secondary Antibody, Alexa Fluor Plus 594, for 1 h. Following this, the samples were stained with DAPI. Images were captured using the Leica DM6B system and Zeiss LSM 880 confocal microscope.

### Measurement of mouse serum PPi and phosphorus (Pi)

To measure mouse serum PPi and Pi, we collected blood and let it clot at room temperature for 30 min. Centrifuge the clotted blood at 3000*g* for 15 min to separate the serum, then carefully remove the serum and store it at − 80 °C. Detection of PPi using Pyrophosphate Assay Kit (ab112155, Abcam), when ready to assay, equilibrates all reagents to room temperature and prepares the reaction mix as per the kit instructions. Add 50 µL each of standards, samples, and reaction mix to a 96-well plate, incubate for 30 min, monitor fluorescence at Ex/Em = 316/456 nm, and calculate PPi activity using the standard curve. The detection of Pi uses a serum inorganic phosphorus detection kit (BB-47426, Bestbio), which combines inorganic phosphorus with ammonium molybdate to generate ammonium phosphomolybdate. The absorbance at 325–340 nm is detected by an ELISA reader, and the inorganic phosphorus content is calculated according to the formula.

### RNA extraction and RT-qPCR

Cells were harvested for gene expression analysis at 14 and 28 days of osteogenic induction. For RNA extraction, the RNeasy Animal RNA Extraction Kit (Beyotime, Shanghai, CHN) was used, followed by the Primescript™ RT Master Mix (perfect real-time) cDNA Synthesis Kit (Takara Biomedical Technology, Beijing, CHN), which converted 400 ng of RNA to cDNA. qRT-PCR was performed using PowerUp™ SYBR™ Green Master Mix (Thermo Fisher Scientific, USA) on a Real-Time System (Bio-Rad). Each reaction mixture was 10 μl and contained 2 μl of 5 ng/μl cDNA, 5 μl of 2× PowerUp SYBR Green Master Mix, 2 μl of nuclease-free water, and 0.5 μl each of 10 μM forward and reverse primers. The following cycle conditions were applied: 50 °C for 2 min and 95 °C for 2 min followed by 44 cycles of 15 s at 95 °C and 1 min at 60 °C. The specific primers used in this study were designed using Primer 6.0 software (Applied Biosystems, Foster City, CA, USA), and the sequences are provided in the supplementary table. The data were analyzed using the 2^−ΔΔ*Ct*^ algorithm.

### Protein extraction and WB analysis

Following treatment, cells were promptly placed on ice and then washed thrice with ice-cold PBS. Cells were lysed with RIPA lysis buffer (Boster, Wuhan, China), containing 1% HaltTM Protease Inhibitor Cocktail (Thermo Fisher Scientific, Waltham, MA, USA) and 1% PMSF (Boster). Subsequently, the cells were sonicated for 30 s using the Sonics CVX130 (Newtown, MA, USA). After centrifugation at 12000*g* for 10 min at 4 °C, protein isolation was carried out. Protein extracts were separated using NuPAGETM 4–12% Bis–Tris gels (Invitrogen), followed by electroblotting to PVDF membranes (Invitrogen). The membranes were water-rinsed and blocked with 5% nonfat dry milk in TBST (Leagene, Beijing, China) for 1 h at room temperature with continuous agitation. Thereafter, the membranes were incubated overnight at 4 °C in TBST with various antibodies including ENPP1, RUNX2, SP7, COL2A1, SOX9, SHH, PTCH, GLI1, p21, p16, and GAPDH. Post-incubation, the membranes were washed thrice with TBST. Goat anti-rabbit HRP (1:4000, ab205718, Abcam) was applied and incubated at room temperature for 1 h. After washing three times with TBST, immunoblots were visualized utilizing an ECL reagent (Beyotime). This experiment was replicated three times.

### Statistical analysis

The results were quantified based on at least three independent experimental groups and presented as the mean ± standard deviation (SD). This study was statistically analyzed using GraphPad Prism 9 software (GraphPad Software Inc., San Diego, CA, USA). Two-tailed Student's t-test or one-way analysis of variance (ANOVA) was used for comparing groups, followed by Bonferroni's multiple comparison test. (*P < 0.05, **P < 0.01, ***P < 0.001, ****P < 0.0001), and *P* < 0.05 was considered significant.

## Results

### ***Enpp1***^***flox/flox***^***/EIIa-Cre*** mice: construction, breeding, and gene identification

The mouse model was constructed and bred as shown in Fig. 1A, the Enpp1^CKI/+^ mice were crossed with EIIa-Cre tool mice to obtain Enpp1^CKI/+^/EIIa-Cre mice. Subsequently, hybridization was carried out in Enpp1^CKI/+^/EIIa-Cre mice to obtain *Enpp1*^*flox/flox*^*/EIIa-Cre* mice. As experimental mice with the following tail gene identification results (Fig. 1C), the 335-bp bands were amplified using F5R5 primers for wild-type (WT) mice, and the 447-bp bands were amplified using EIIa primers for EIIa-Cre mice. The heterozygous Enpp1^CKI/+^/EIIa-Cre mice had a 641-bp band after F4R4 amplification, a 335-bp band after F5R5 primer amplification, a 447-bp band after EIIa forward and reverse primer amplification, and a 512-bp band after F4R6 primer amplification. In contrast, the *Enpp1*^*flox/flox*^*/EIIa-Cre* mice only had a 512-bp band following the amplification of the F4R6 primer, and the EIIa forward and reverse primers amplified a 447-bp band. Sequencing after amplification using primer F4R6 suggested sequence agreement with the construct, that is, 568 sites (GGA to TGA) mutation (Fig. 1D).

### Imaging and pathological assessment of the spine, knees, Achilles tendons, and hips of ***Enpp1***^***flox/flox***^***/EIIa-Cre*** and WT mice with increasing age

**Fig. 2:**
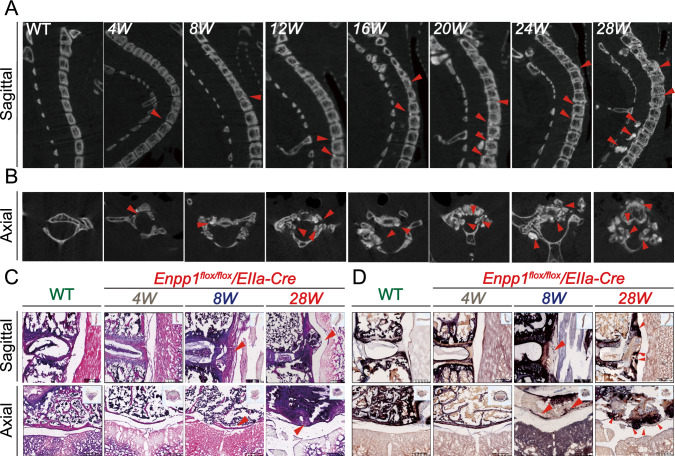
Representative micro-CT and histopathology of the spine. **A** Sagittal image. **B** Axial image at the corresponding time point, red arrows indicate ossification of ligaments, intervertebral discs, and formation of osteophytes in articular processes. **C**-**D** Representative HE and ALP staining of the spine from mice at week 4.8.28. At 28 weeks, red arrows indicate the spinal cord was severely compressed. Scale bars = 200 um, *n* = 6

The results demonstrated that mice of all *Enpp1*^*flox/flox*^*/EIIa-Cre* genotypes consistently exhibited varying degrees of heterotopic ossification in the spine, knee, Achilles tendon, and hip joints as they aged (*n* = 6). As shown in Fig. 2A, from week 4 (4 W), *Enpp1*^*flox/flox*^*/EIIa-Cre* mice revealed slight ossification hyperplasia in the upper and lower endplates originating from the posterior longitudinal ligament and the intervertebral disc. As the ossified area grew larger from 12 to 28 weeks, it gradually compressed the spinal canal. There was significant OPLL at the spinal junction of the cervical and thoracic segment by 28 weeks (28 W) in *Enpp1*^*flox/flox*^*/EIIa-Cre* mice, accompanied by severe bone formation and bridging ankylosis at the junction. According to the axial section, the ossification site of the spine is located behind the posterior longitudinal ligament. In addition, numerous ossification foci are formed in the bilateral articular processes towards the spinal canal (Fig. 2B). Meanwhile, ligamentous ossification remained unobserved in WT mice (sFig. 1A). In addition, we supplemented the CT of knees and Achilles tendons with 4 W to 28 W in WT mice, which showed there is no obvious ectopic ossification formation and degeneration of knees (sFig. 1B–C).

HE staining showed progressive ossification of the posterior longitudinal ligament of the spine in mice starting at 8 weeks, with the reactive proliferation of fibro ring chondrocytes. In contrast, the PLLs of WT mice showed normal dense collagen fibers in which fibroblasts were located. At 28 weeks, mice showed generalized dysplasia and hypertrophic chondrocytes in lacunar structures with large, ossified masses compressing the posterior spinal cord. ALP staining revealed attachment of epiphyseal plate chondrocytes and osteoblasts to the posterior longitudinal ligament starting at 8 W. Ligament staining was evident at 28 W, suggesting calcification deposited at the junction of the intervertebral disc and posterior longitudinal ligament (Fig. 2C and D).

At 12 weeks (12 W), mice gradually developed articular surface unevenness in the knee joint with knee osteophytes, knee degeneration was evident, as well as HO of the knee surface (sFig. 2A), during which a gradual decrease in femoral and tibial bone mass and sparse bone trabeculae could be observed. In the knee joint of 8W *Enpp1*^*flox/flox*^*/EIIa-Cre* mice, tissue staining suggested the proliferation of chondrocytes and osteoblasts above and below the patellar ligament, accompanied by proliferation and calcification of osteo-blasts in the joint meniscus by 28 W. Alcian Blue & Nuclear Fast Red staining suggested severe degeneration of the joint surface, late loss of subchondral bone and its replacement by fat and intramedullary tissue, and thinning of the bone cortex, suggesting late bone remodeling (sFig. 2B–2D). To quantify the degree of osteoporosis in mice, we analyzed the bone volume of the hip (sFig. 3A-F), and the results suggested that BV/TV, BS/BV, Tb.N, and Tb.Th all decreased significantly with age, while Tb. Sp increased significantly with age, suggesting that *Enpp1*^*flox/flox*^*/EIIa-Cre* mice had significant osteoporosis with age.

**Fig. 3:**
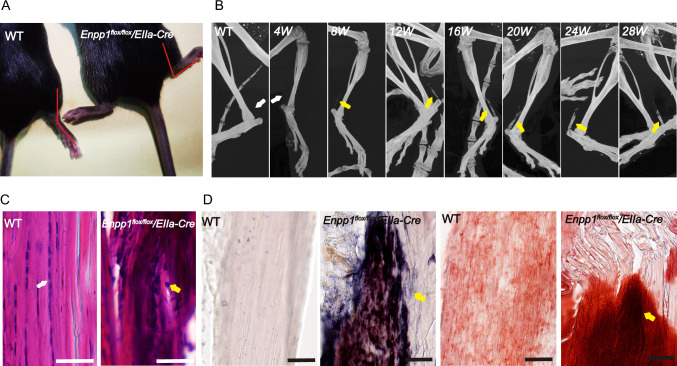
Representative micro-CT and histopathology of the Achilles. **A** Due to ossification of the Achilles' tendons and reduced ankle range of motion, *Enpp1 *^*flox/flox*^*/EIIa-Cre* mice exhibit spastic ankles compared with WT mice. **B** The Achilles tendons of the mice gradually aged. 4W, 8W, 12W, 16W, 20W, 24W, and 28W *Enpp1 *^*flox/flox*^*/EIIa-Cre*, compared to WT mice (8W) Achilles tendon micro-CT image. Yellow arrows indicate the ossification formation of the Achilles tendon. **C-D** HE, ALP, and alizarin red staining in mice. Yellow arrows show positively stained tissues, Scale bars = 50 um, *n* = 6

At eight weeks, mice began to show heterotopic ossification of the Achilles tendon (Fig. 3B). HE and ALP staining suggested that the Achilles tendon of *Enpp1*^*flox/flox*^*/EIIa-Cre* mice also showed distinct areas of ossification surrounded by chondrocyte-like cells, the cartilaginous matrix in the middle part of the tendon, and many gaps at the end of the tendon, with an increase in osteoblasts and chondrocyte, mostly concentrated at the junction between the Achilles tendon and the heel bone. Alizarin red staining confirmed the formation of calcium deposits in the ossified area (Fig. 3C and D).

### Cells from the spinal ligaments are isolated, identified, and osteogenically induced

**Fig. 4:**
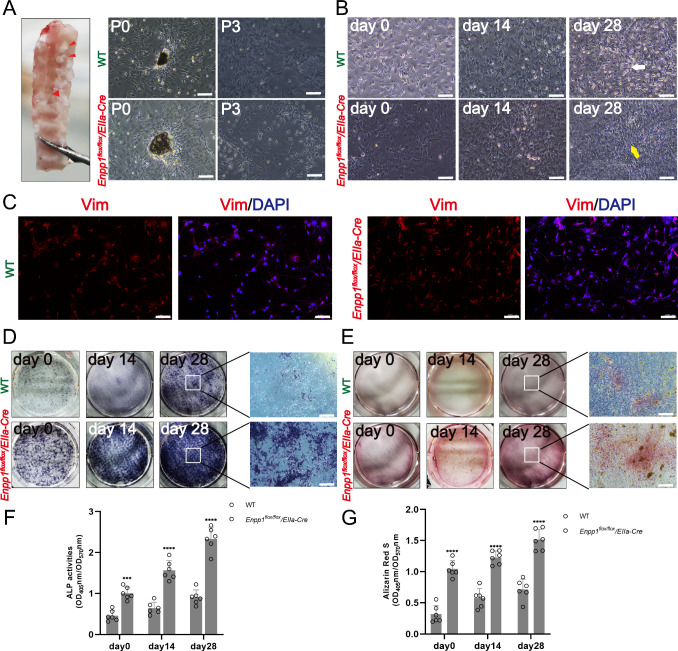
The posterior longitudinal ligament of mice was taken for culture as indicated and osteogenic induction. **A**-**B** Culture and osteogenic induction of posterior longitudinal ligament cells in mice. **C** Vimentin staining identified that most of the cells were fibroblasts. **D**-**E** 0, 14, 28 days of osteogenic induction by ALP and alizarin red staining, from 0 to 28 days. (*n* = 6), scale = 100 um

Firstly, we obtained the posterior longitudinal ligament (PLL) from the spines of mice and successfully isolated the primary cells. The P3 generation was used in the experiment (Fig. 4A). After osteogenesis induction, we observed that the cells at 14 and 28 days showed a relatively polygonal shape; at the same time, the WT group retained most of its fibroblast phenotype (Fig. 4B). Cells were confirmed to be fibroblasts by vimentin immunofluorescence staining (Fig. 4C). After 14 and 28 days of induction, ALP and Alizarin red staining were stronger and deeper than those in WT mice (Fig. 4D–G).

### Enpp1 deficiency alters cell differentiation in spinal ligaments and accelerates knee joint degeneration

**Fig. 5:**
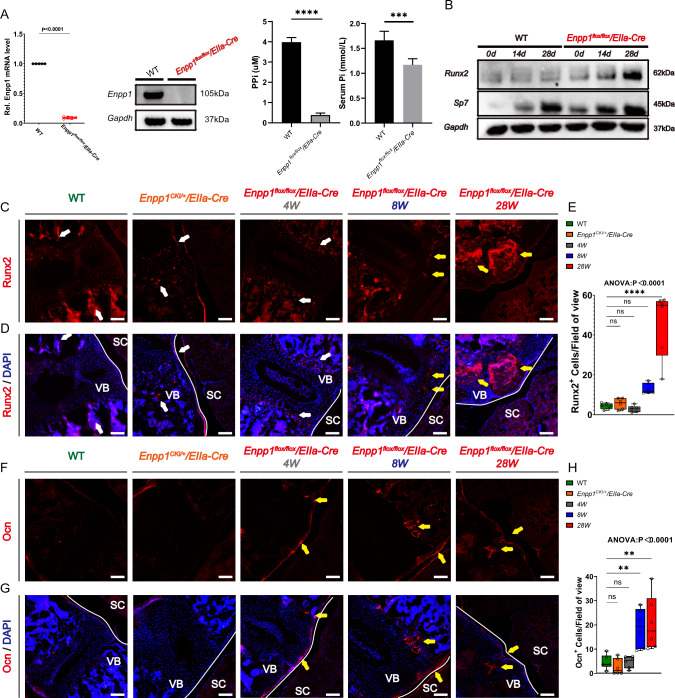
The osteogenic signal of spinal ligament and intervertebral disc in *Enpp1*^*flox/flox*^*/EIIa-Cre* mice increased. **A** qRT-PCR, WB, serum PPi, and Pi level were used to determine Enpp1 mRNA and protein expression, *n* = 5. **B** WB was used to determine Runx2 and Sp7 protein expression levels. **C**-**G** Immunofluorescence of Runx2, Ocn, Opn, and Ibsp in PLL of *Enpp1*^*flox/flox*^*/EIIa-Cre*, Enpp1^CKI/+^/EIIa-Cre, and WT mice at 4, 8, and 28 weeks. yellow arrows represent high expression areas in each group, white arrows represent blank control areas, *n* = 6, Scale bars = 250 um, vertebra (VB), spinal cord (SC)

**Fig. 6:**
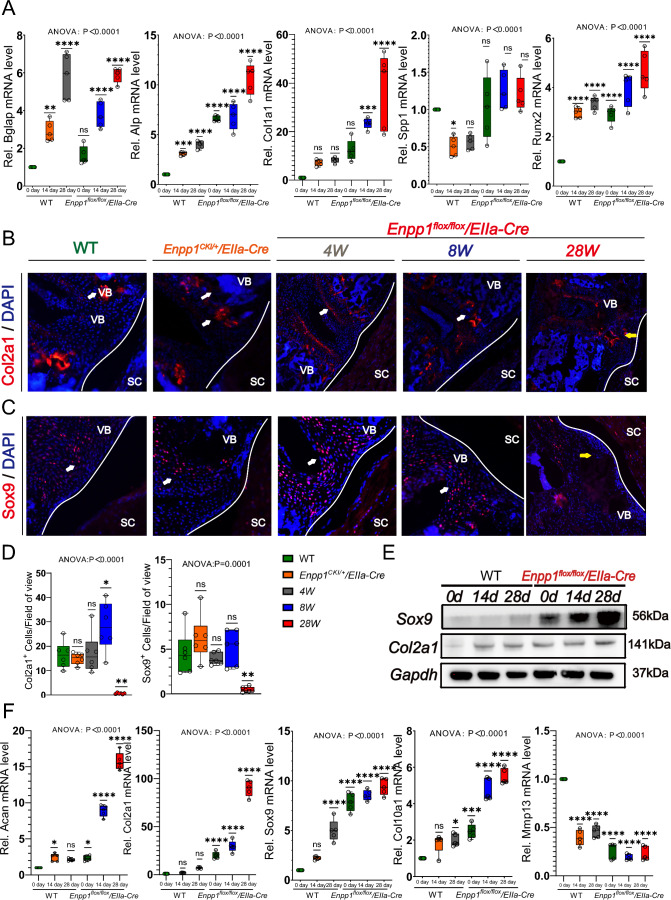
Changes of chondrogenic signal distribution in spinal ligaments and intervertebral discs of mice. **A** The mRNA expression of Bglap, Alp, Col1a1, Spp1, and Runx2 was determined by qRT-PCR. **B**-**D** Immunofluorescence of Col2a1 and Sox9 in PLL of *Enpp1*^*flox/flox*^*/EIIa-Cre*, Enpp1^CKI/+^/EIIa-Cre and WT mice at 4, 8, and 28 weeks, the yellow arrow indicates that Col2a1 expression of Enpp1 mice in late stage is gradually sparse and uneven, while Sox9 is almost not expressed at 28 weeks, white arrows represent blank control areas, vertebra (VB), spinal cord (SC), Scale bars = 250 um, *n* = 6. **E** The protein expression of Col2a1 and Sox9 was determined by WB. (F) The mRNA expression of Acan, Col2a1, Sox9, Col10a1, and Mmp13 was determined by qRT-PCR, *n* = 5

We performed immunofluorescence staining for Runx2, Ocn, Opn, Ibsp, Col2a1, and Sox9 in spine sections from 4 W, 8 W, and 28 W *Enpp1*^*flox/flox*^*/EIIa-Cre*, Enpp1^CKI/+^/EIIa-Cre, and WT mice. Because ossification occurred at 8 W, we used 8 W *Enpp1*^*flox/flox*^*/EIIa-Cre*, and WT mouse posterior longitudinal ligament cells for culture and osteogenesis induction. Finally, the osteogenic cartilage expression and osteogenic potential of both mice were detected by quantitative real-time PCR (qRT-PCR) and Western blotting (WB). We confirmed that Enpp1 was not expressed in *Enpp1*^*flox/flox*^*/EIIa-Cre* mice at the mRNA and protein levels (Fig. 5A). Besides, compared with WT mice, the PPi value of serum in *Enpp1*^*flox/flox*^*/EIIa-Cre* mice was lower (p < 0.0001). Besides, compared with WT mice (1.66 ± 0.18 mmol/L), the Pi value of serum in *Enpp1*^*flox/flox*^*/EIIa-Cre* mice (1.17 ± 0.12 mmol/L) was lower (p < 0.001) (Fig. 5A). After 28 days of osteogenic induction by ligament cells, *Enpp1*^*flox/flox*^*/EIIa-Cre* mice showed higher osteogenic activity compared to WT mice (mRNA level, Bglap, Runx2, Alp, Col1a1; protein level, Runx2, Sp7) (Figs. 5B, 6A).

Runx2 expression was detected in the vertebral bodies of the spine in WT and Enpp1^CKI/+^/EIIa-Cre mice and not in the intervertebral discs. *Enpp1*^*flox/flox*^*/EIIa-Cre* mice from 4 to 8 weeks also expressed only in the upper and lower endplates, indicating that it was not expressed around the posterior longitudinal ligament before the maturation of HO. Whereas high Runx2 expression was detected in the ectopic ossified tissue of the posterior longitudinal ligament in 28 W *Enpp1*^*flox/flox*^*/EIIa-Cre* mice (Fig. 5C). Ocn and Ibsp were similarly expressed in the spine; both were highly expressed in the anterior and posterior longitudinal ligaments of the vertebral body, and their expression increased with increasing age in mice, compared with the WT and the Enpp1^CKI/+^/EIIa-Cre mice, the expression of Ocn in the superior and inferior spinal endplates with the posterior longitudinal ligament was increased in *Enpp1*^*flox/flox*^*/EIIa-Cre* mice. As for Ibsp, components of the mineralized matrix seem to be formed. May be important for cell–matrix interactions. Ibsp was also only expressed in the upper and lower middle plates and posterior margins of the vertebral bodies at the early stage and was highly expressed in the heterotopic ossified parts at the late stage (Fig. 5D, F). Opn was uniformly expressed in the upper and lower endplates of the vertebral body in WT mice, while it was barely expressed in the upper and lower endplates in *Enpp1*^*flox/flox*^*/EIIa-Cre* mice at 4 weeks, unevenly expressed at 8 weeks, and highly expressed at ectopic ossification sites at 28 W (Fig. 5E).

In contrast, the early cartilage-related indices Col2a1 and Sox9 expression in the spine are similar. Unlike joints, Col2a1 and Sox9 expression in the spine does not decrease with age in the early stages but increases. 4 W and 8 W *Enpp1*^*flox/flox*^*/EIIa-Cre* mice expressed Col2a1 in similar locations to WT mice, both in the upper and lower endplates of the vertebral body, while late 28 W mice expressed more Col2a1 on the side of the bone below the endplate (Fig. 6B). Sox9 was expressed in the upper and lower endplates and in the nucleus pulposus; late 28 W mice expressed Sox9 only in the nucleus pulposus, with almost no expression in the upper and lower endplates, while its expression was significantly higher in the vertebral body than in other groups of mice (Fig. 6C and D). In the mouse knee, Col2a1 and Sox9 expression decreased with increasing age (sFig. 2E-F), which is consistent with previous reports. Col2a1 was normally expressed in the subchondral bone and around the growth plate of the knee joint. There was no significant difference between 8W mice and WT mice, but the joint surface was uneven at the end of 28 W, and the expression of Col2a1 decreased significantly. Sox9 was mostly expressed on the surface of the knee joint, which was similar to the expression trend of Col2a1. A similar level of expression was observed in the early stage of the study as in WT mice, however, the level significantly decreased by 28 W. For WT mice, there were no significant differences in Runx2, Ocn, Opn, Ibsp, Col2a1, and Sox9 expression at the intervertebral disc or in the posterior longitudinal ligament region (sFig. 4). After 28 days of osteogenesis induction, chondrogenic indicators (mRNA level, Acan, Col2a1, Sox9; protein level, Col2a1, Sox9) were significantly elevated, and the late mast chondrocyte marker Col10a1 was increased, while Mmp13 was decreased (Fig. 6E and F). These results suggested that the occurrence of ectopic ossification is pathogenic based on the hypertrophy of chondrogenic cells.

### Assessment of growth, aging, and behavioral function in ***Enpp1***^***flox/flox***^***/EIIa-Cre*** mice

**Fig. 7:**
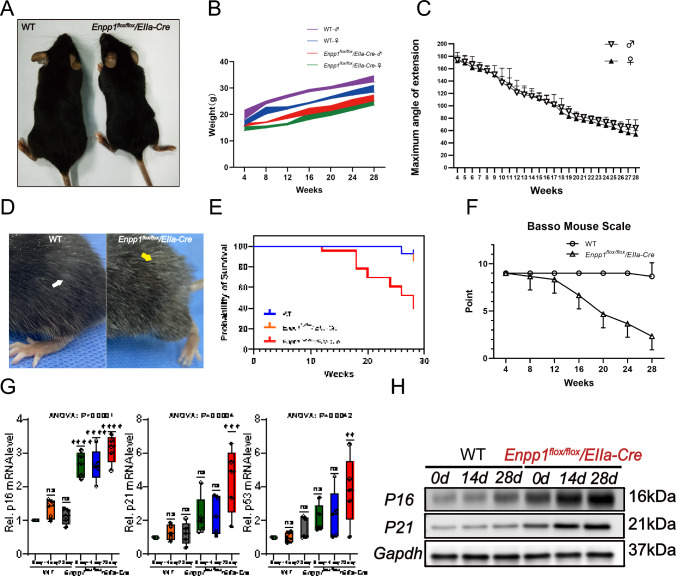
Comparison of development and aging-related factors between *Enpp1*^*flox/flox*^*/EIIa-Cre* and WT mice. **A** E *Enpp1*^*flox/flox*^*/EIIa-Cre* mice are stunted, and lighter than WT mice. **B** Results of the evaluation of mice aged 4 weeks to 28 weeks indicate that male and female *Enpp1*^*flox/flox*^*/EIIa-Cre* mice have a smaller body weight than WT mice at the same age. **C** An analysis of the ROM of ankle joints. **D** *Enpp1*^*flox/flox*^*/EIIa-Cre* mice exhibited premature cutaneous hair aging, with a significant increase in white hair occurrence at 28 weeks compared to age-matched WT mice. **E** The survival probability of *Enpp1*^*flox/flox*^*/EIIa-Cre* mice is lower than that of WT mice and Enpp1^CKI/+^/EIIa-Cre mice of the same age group. **F** Based on BMS scores, the open field walking ability is determined. (*n* = 3 mice/group). **G**–**H** RT-qPCR was used to determine p16, p21, and p53 mRNA expression levels. WB was used to determine p16 and p21 protein expression levels

*Enpp1*^*flox/flox*^*/EIIa-Cre* mice grew more slowly than WT mice and females and males weighed less than mice of the same age (Fig. 7A and B). The results showed that Enpp1 deficiency with age resulted in limited joint mobility, which correlated with HOTL progression. A potential impact of progressive HO of the Achilles tendon with increasing age in *Enpp1*^*flox/flox*^*/EIIa-Cre* mice was investigated by monitoring their ankle joint range of motion (ROM) weekly between 4 and 28 weeks of age (Fig. 3A). As shown in Fig. 7C, the ROM of *Enpp1*^*flox/flox*^*/EIIa-Cre* mice decreased significantly with age, and at week 28 W, the ankle extension angle was severely limited in *Enpp1*^*flox/flox*^*/EIIa-Cre* mice, whereas it decreased to a lesser extent in WT mice (ROM close to 180 degrees). The *Enpp1*^*flox/flox*^*/EIIa-Cre* mice exhibit an aging phenotype as well as a slower developmental rate. At 28 W, *Enpp1*^*flox/flox*^*/EIIa-Cre* mice developed earlier aging, with more white hair evident (Fig. 7D). Moreover, *Enpp1*^*flox/flox*^*/EIIa-Cre* mice had a lower survival rate than WT and Enpp1^CKI/+^ /EIIa-Cre mice beyond 12 W of age (Fig. 7E). Spinal cord function in *Enpp1*^*flox/flox*^*/EIIa-Cre* mice was assessed by the Basso Mouse Scale (BMS) score every 4 weeks (Fig. 7F). From 8 to 12 weeks of age, *Enpp1*^*flox/flox*^*/EIIa-Cre* mice had significantly lower BMS scores than WT mice. In addition, we examined the senescence of ligament fibroblasts in WT and *Enpp1*^*flox/flox*^*/EIIa-Cre* mice, and qRT-PCR results showed that *Enpp1*^*flox/flox*^*/EIIa-Cre* mice ligament cells expressed higher levels of p16, p21, and p53, and the expression increased with osteogenic induction time. For p16 and p21, WB results were like those obtained by qRT-PCR (Fig. 7G and H).

### Loss of Enpp1 leads to upregulation of Hh signaling during spine development

**Fig. 8:**
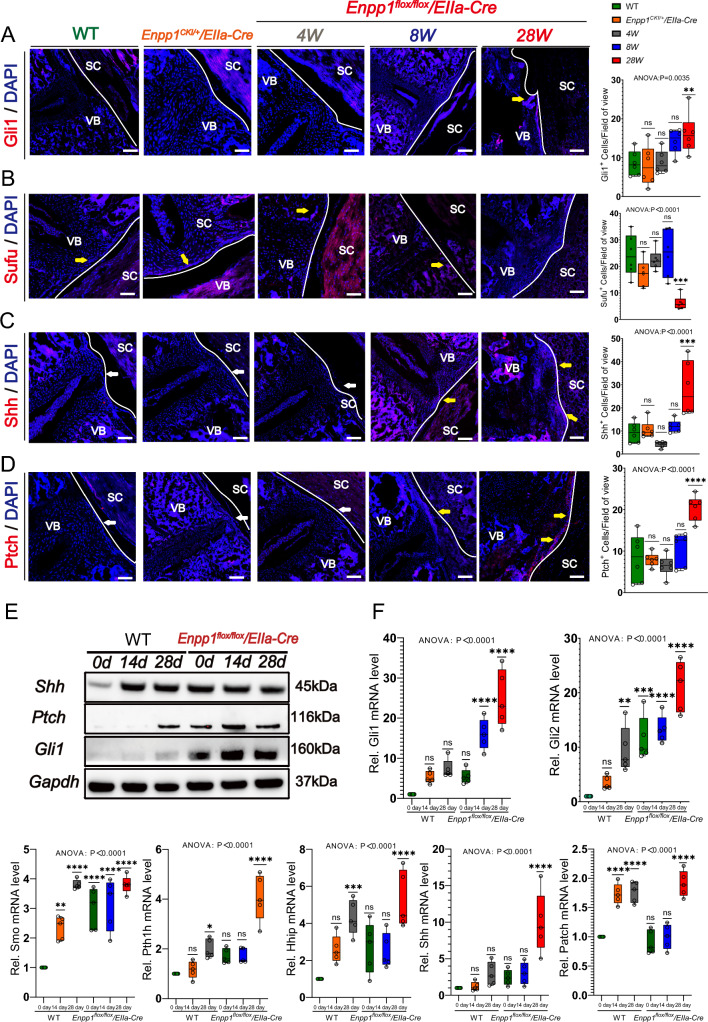
The hedgehog signal of spinal ligament and intervertebral disc in *Enpp1*^*flox/flox*^*/EIIa-Cre* mice increased. **A**-**D** Immunofluorescence of Gli1 and Sufu in PLL of *Enpp1*^*flox/flox*^*/EIIa-Cre*, Enpp1^CKI/+^/EIIa-Cre, and WT mice at 4, 8, and 28 weeks, yellow arrows represent positive expression areas in each group, white arrows represent blank control areas. vertebra (VB), spinal cord (SC). Scale bars = 250 um, *n* = 6. **E** WB of Shh, Ptch, and Gli1 in PLL-derived fibroblasts with and without osteogenic induction, *n* = 3. **F** qRT-PCR analysis of Gli1, Gli2, Shh, and Smo, Hhip, Patch1, Pth1h in PLL-derived fibroblasts with and without osteogenic induction. Data are presented as the mean ± SD, *n* = 5

*Enpp1*^*flox/flox*^*/EIIa-Cre* and WT spines were further examined by immunofluorescence staining for Ptch, Gli1, and Shh protein expression in 4 W, 8 W, and 28 W mice. The PLL of *Enpp1*^*flox/flox*^*/EIIa-Cre* contained more Ptch-positive, Gli-positive, and Shh-positive fibro-blasts compared with WT mice, In WT, Enpp1^CKI/+^/EIIa-Cre mice as well as early 4W, 8W *Enpp1*^*flox/flox*^*/EIIa-Cre* mice, there is a minor expression of Gli1, Shh, Ptch around the vertebral bodies and no expression in the posterior longitudinal ligament and discs, whereas, in late 28 W *Enpp1*^*flox/flox*^*/EIIa-Cre* mice, ectopic ossification is seen around the periphery and high expression in the posterior longitudinal ligament. In contrast, Sufu was barely expressed around the HO region of the spine (Fig. 8A–D), and the expression was progressively enhanced with increased PLL ossification. The expression of Ptch, Gli1, and Shh in fibroblasts from the PLL of the *Enpp1*^*flox/flox*^*/EIIa-Cre* mice was also significantly increased by WB, and expression was further enhanced after osteogenesis induction (Fig. 8E). Expression of genes associated with the Hh pathway, such as Shh, Gli1, Gli2, Smo, Hhip, and Pth1h, was increased in fibroblasts from the PLL of *Enpp1*^*flox/flox*^*/EIIa-Cre* mice compared with WT mice (Fig. 8F). For WT mice, there were no significant differences in Gli1, Sufu, Shh, and Ptch expression at the intervertebral disc or in the posterior longitudinal ligament region (sFig5). In conclusion, the findings suggest that the cells undergo chondrohypertrophy and osteogenic differentiation due to the high activity of Hh signaling in PLL.

## Discussion

At present, there are still many disputes about the osteogenic mechanism of HOTL. There is increasing evidence that HOTL is associated with endochondral ossification, with high expression of osteogenic and chondrogenic markers in tissue cells, which also supports the pathogenesis of OPLL to some extent. The study primarily established a model of spinal ectopic ossification, shedding light on its characteristics and the genetic mechanisms involved. E *Enpp1*^*flox/flox*^*/EIIa-Cre* mice were constructed as a basis for our future studies, and all phenotypes can be used as references. Various tool mice will be used in future studies to hybridize with them, targeting tissue-specific mutations or expressing mutations at a specific time. It will be important for us to further characterize the Enpp1 function and HO of ligaments.

First, we generated *Enpp1*^*flox/flox*^*/EIIa-Cre* mice using CRISPR-Cas9 technology, we mainly verified the feasibility of the model at this stage. Therefore, we used EIIa-Cre mice as tool mice, Cre recombinase in nearly in all tissues (whole body EIIa knockout). In the next stage, we will use different tool mice to carry out conditional knockout in time and space. These mice serve as an ectopic ossification model similar to the mice. Enpp1^CKI/ +^ is a conditional knock-in mouse in which embryonic direct mutation lethality is also avoided. Phenotypically, *Enpp1*^*flox/flox*^*/EIIa-Cre* mice exhibit an ossified spine and the onset of Achilles tendon abnormalities at 4–8 weeks of age. Notably, these mice demonstrate significant hindlimb dysfunction by 8 weeks, which occurs earlier compared to the mice. The site of spinal cord ossification is slightly different in the mice compared with *Enpp1*^*flox/flox*^*/EIIa-Cre* mice. Although some ossification foci are also observed in the posterior longitudinal ligament, there are many spherical ossifications around the small articular process, and the spinal cord is more severely compressed, which is also an excellent model of chronic spinal cord compression. The ttw mice were mostly ossified just posterior to the centralized posterior longitudinal ligament with many flat-type ossification foci, which may result from different mouse species backgrounds and gene mutation patterns.

Enpp1-generated PPi inhibits bone mineralization and soft tissue calcification by binding to nascent hydroxyapatite crystals, thereby preventing further growth of these crystals. Various Enpp1 mutations have been identified in humans, but the phenotypes of patients carrying different Enpp1 mutations include OPLL, GACI and hypophosphatemic rickets, and different phenotypes occur with Enpp1 mutations in mice, which also include HO and impaired glucose tolerance. Osteoporotic mice deficient in Enpp1 develop a variety of progeroid symptoms [35–37]. In addition, we observed that *Enpp1*^*flox/flox*^*/EIIa-Cre* mice developed severe knee degeneration, osteoporosis, and a progressive reduction in spinal cord function and ROM in the ankles as they aged. We also confirmed Enpp1 as an anti-aging factor that regulates calcium balance. Mice lacking Enpp1 exhibited a decrease in average lifespan and early signs of hair whitening, highlighting its importance. At the cellular level, there was an increase in the expression of cell cycle markers associated with aging, further intensified by osteogenic induction. This underscores the complex interplay between Enpp1, calcium regulation, and the aging process.

In the spinal region, the trend of osteogenic expression was also consistent, with Ocn, Runx2, and Ibsp being mostly expressed in the vertebral body in the WT and Enpp1 groups at the early stage (4 W, 8 W) and highly expressed at the site of ectopic ossification at 28 W. Both Ocn and Ibsp are tightly bound to hydroxyapatite and appear to form an integral part of the mineralized matrix, whereas Runx2 is a transcription factor involved in osteoblast differentiation and skeletal morphogenesis and is essential for osteoblast maturation and both intramembranous and endochondral ossification [38–40]. This finding suggests that not only the deposition of calcium salts but also osteoblasts are involved in the process of HO. Contrary to the WT group, Opn was uniformly expressed in the upper and lower endplates of the vertebral body, whereas Opn was irregularly expressed in experimental mice, with high expression at 28 days post-ossification of the anterior and posterior longitudinal ligaments. Opn produces a major noncollagenous bone protein tightly bound to hydroxyapatite, which is an integral part of the mineralized matrix and may be important for osteoblast matrix interactions [41]. The qRT-PCR and WB results also confirmed that osteogenic and chondrogenic indices were also significantly enhanced in young mouse spinal fibroblasts after osteogenic induction, and the expression of Col10a1, a marker of mast chondrocytes, was increased compared with WT mice, suggesting that the loss of Enpp1 expression leads to high early chondrocyte expression, whereas it is repressed late, and promotes ectopic ossification.

Various studies have also suggested that upregulation of Hh signaling activity in the absence of Enpp1 may contribute to ectopic calcification and OA of the knee in different tissues [25, 42]. Enpp1 was expressed at a significantly higher level in OA meniscal cells than in normal meniscal cells [43, 44]. Furthermore, ectopic activation of Hh signaling leads to long bone synovial joint osteoarthritis. Our study shows that, unlike articular expression, Col2a1 expression in cartilage and endplates did not decrease with age in the experimental group. Sox9 expression was progressively late from cartilage and nucleus pulposus to confined to the nucleus pulposus, while expression was increased in the vertebral body, it was suggested that chondrocytes play an important role in the HO of spinal ligaments.^31^ Additionally, Enpp1 deficiency-induced ossification in mouse spinal ligaments and micro articular synapses is an active process of early chondrocyte hypertrophy and late osteogenesis, a process that activates Hh signaling. The expression of Shh, Ptch, and Gli was higher in the spinal ligaments of *Enpp1*^*flox/flox*^*/EIIa-Cre* mice than in WT mice, and the number of ligament cells was significantly higher in *Enpp1*^*flox/flox*^*/EIIa-Cre* mice than in WT mice at both the mRNA and protein levels after induction. In the human OPLL study, Daisuke Sugita demonstrated that the ossification front in OPLL patients contained chondrocytes at different stages of differentiation, including proliferating chondrocytes in the fibrocartilage zone, hypertrophic chondrocytes around the calcification front, and apoptotic chondrocytes near the ossification zone. Cultured cells from OPLL tissue ex-pressed significantly higher levels of Ihh, PTHrP, and Sox9 than non-OPLL cells, suggesting that overexpression of Ihh signaling promotes aberrant chondrocyte differentiation during cartilage ossification, enhances bone formation in OPLL, and induces aberrant activation of Ihh and related signaling molecules during cyclic stretch strain [45, 46].

Our research has certain limitations. In this process, we only confirmed that the Hh signal of Enpp1 mice in the spinal HO area and ligament cells is activated, but no evidence is available that the activated signal directly contributes to the progression of OPLL. The relationship between these two needs to be clarified in future research. Finally, our future studies should go deeper to specifically understand the mechanism of action of the Hh signaling pathway, and the results should be further validated in human HOTL samples.

**Fig. 9:**
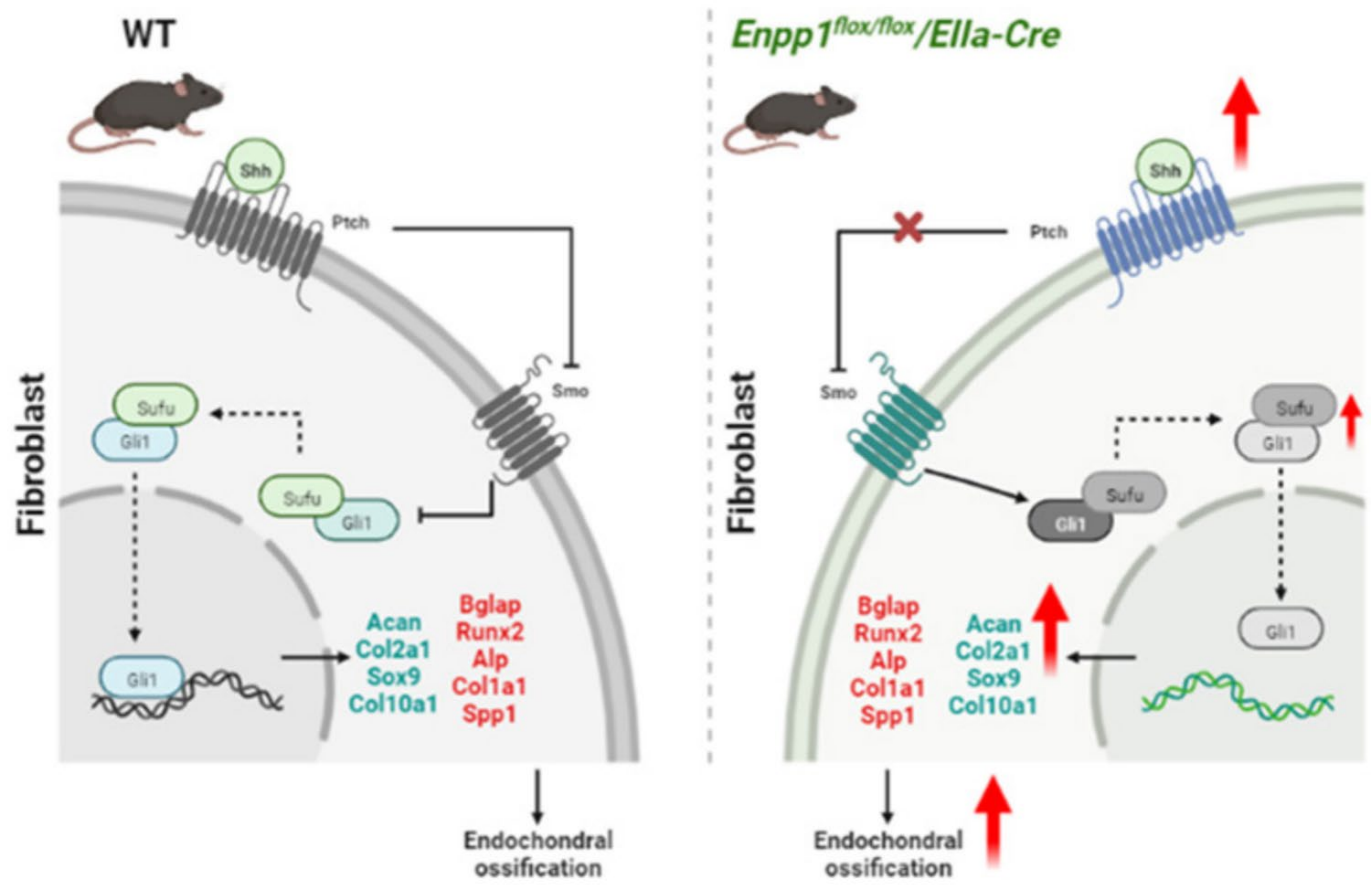
Schematic summary of Hedgehog pathway in WT and *Enpp1*^*flox/flox*^*/EIIa-Cre* mice

In summary (Fig. 9), we constructed mice with HO based on mutations in the Enpp1 gene using CRISPR-Cas9 technology. In *Enpp1*^*flox/flox*^*/EIIa-Cre* mice, the loss of Enpp1 leads to disturbances in bone metabolism in spinal ligaments and Achilles tendons with degeneration of the knee joint, loss of skeletal bone mass, and plays a key role in age-related HOTL, a loss of Enpp1 function accelerates cellular senescence in mice. In this study, as with other types of HO, we found activation of Hh signaling and expression of chondrocyte hypertrophy/osteoblast markers in the spinal ligaments of *Enpp1*^*flox/flox*^*/EIIa-Cre* mice. It was concluded that the calcification in spinal ligaments due to Enpp1 deficiency is not primarily a passive process of hydroxyapatite deposition on the articular surface, but also an active process induced by ectopic Hh signaling that activates novel regulatory mechanisms to promote chondrocyte hypertrophy and osteoblast differentiation. These findings will help us to better understand the complex senescence mechanisms in the development of HOTL.

## Supplementary Information

Below is the link to the electronic supplementary material.

Supplementary file1 (PDF 1529 KB)

## Data Availability

All the data obtained and analyzed during the current study were available from the corresponding authors upon reasonable request.
