# Supplementary material for: Enpp1 mutations promote upregulation of hedgehog signaling in heterotopic ossification with aging
Source: J Bone Miner Metab. 2024 Aug 30;42(6):681–98. doi: 10.1007/s00774-024-01543-1 (PMC11632054; doi:10.1007/s00774-024-01543-1)
Supplement: Supplementary file 1 — Supplementary file1 (PDF 1529 KB) [file 774_2024_1543_MOESM1_ESM.pdf]

## Supplementary figure

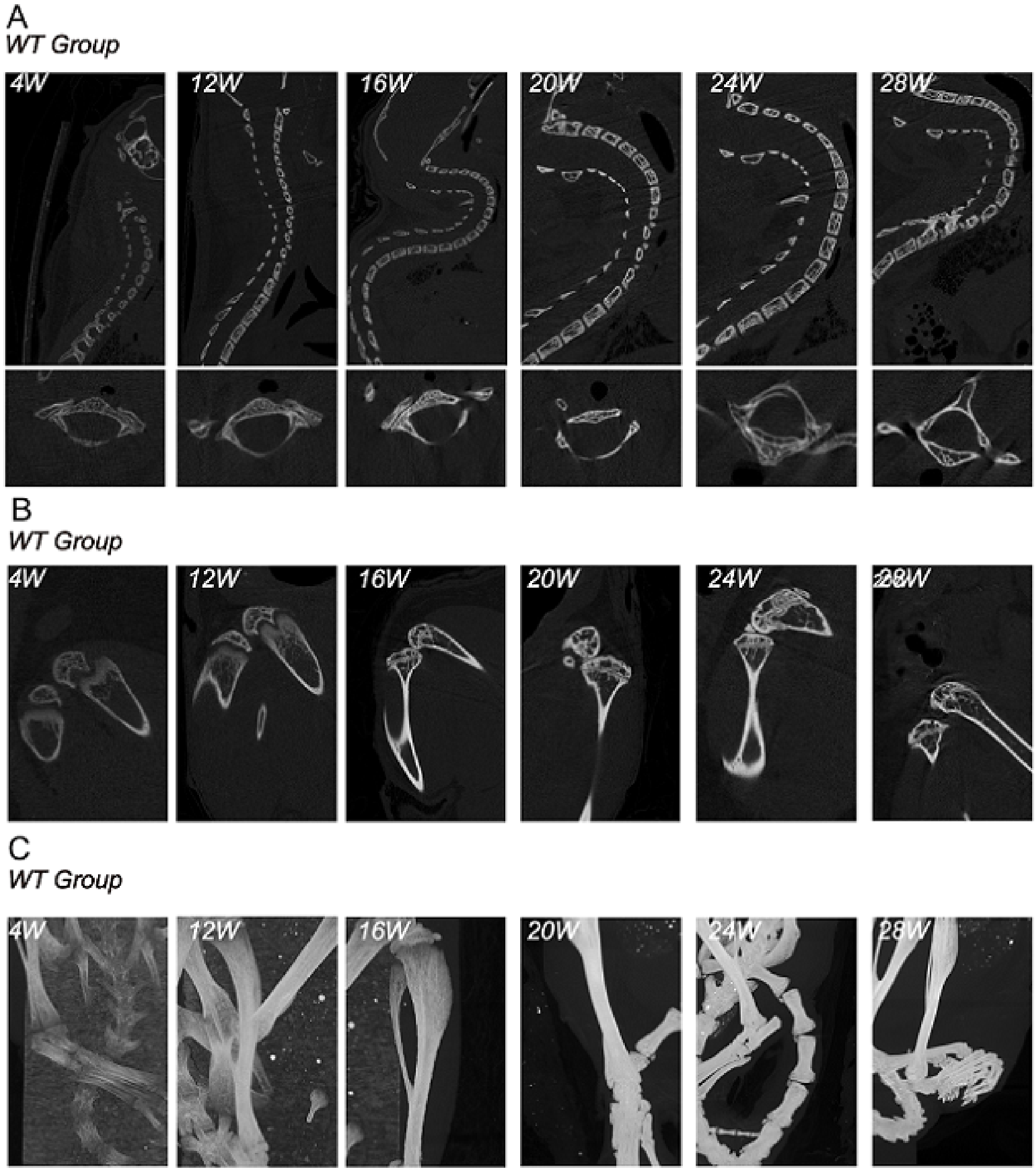


**Supplementary figure 1. The CT scan of WT mice complemented the point-to-point contrast of *Enpp1*$^{flox/flox}$*/ EIIa-Cre* mice.** (A) Cervical spine CT scans of wild-type mice at 4, 12, 16, 20, 24, and 28 weeks showed no ectopic ossification formation. (B) The knees of WT mice degenerated at 4, 12, 16, 20, 24 and 28 weeks.

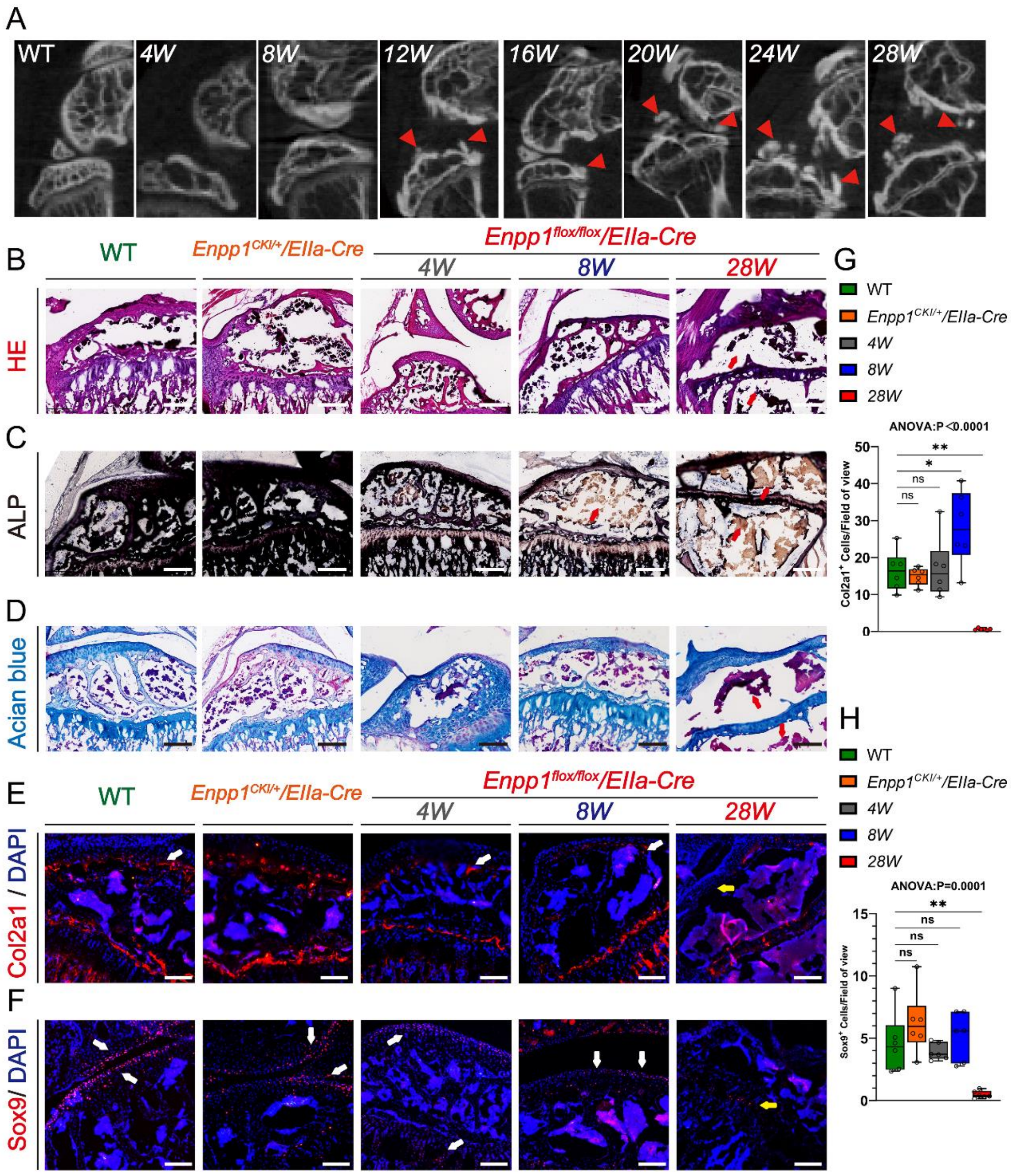


**Supplementary figure 2. Progressive knee degeneration in mice.** (A) Micro CT images of *Enpp1$^{flox/flox}$/ EIIa-Cre*, and WT knee joints at 4, 8, 12, 16, 20, 24, and 28 weeks of age. (B-D) In the knees of mice at different weeks of age for HE and ALP, Alcian blue staining suggests that the cartilage on the surface of the knee joint is degraded. Immunofluorescence staining of sections from mouse knee joints. (E-H) Col2a1, Sox9 immunofluorescence in the knee joints of 4W, 8W, 28W mice.

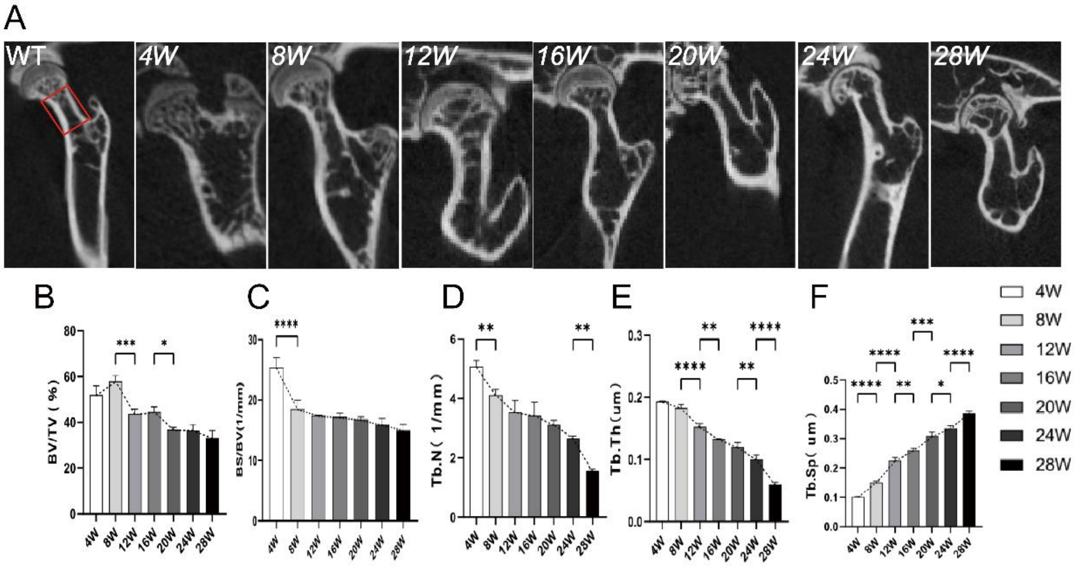


**Supplementary figure 3. Mice have increased hip bone mass changes with age.** (A) To quantify the degree of hip osteopenia, we analyzed the hip bone mass of mice at different ages and selected the same area for mouse hip CT analysis. (B) The results showed that BV/TV, BS/BV, Tb.N, and Tb.

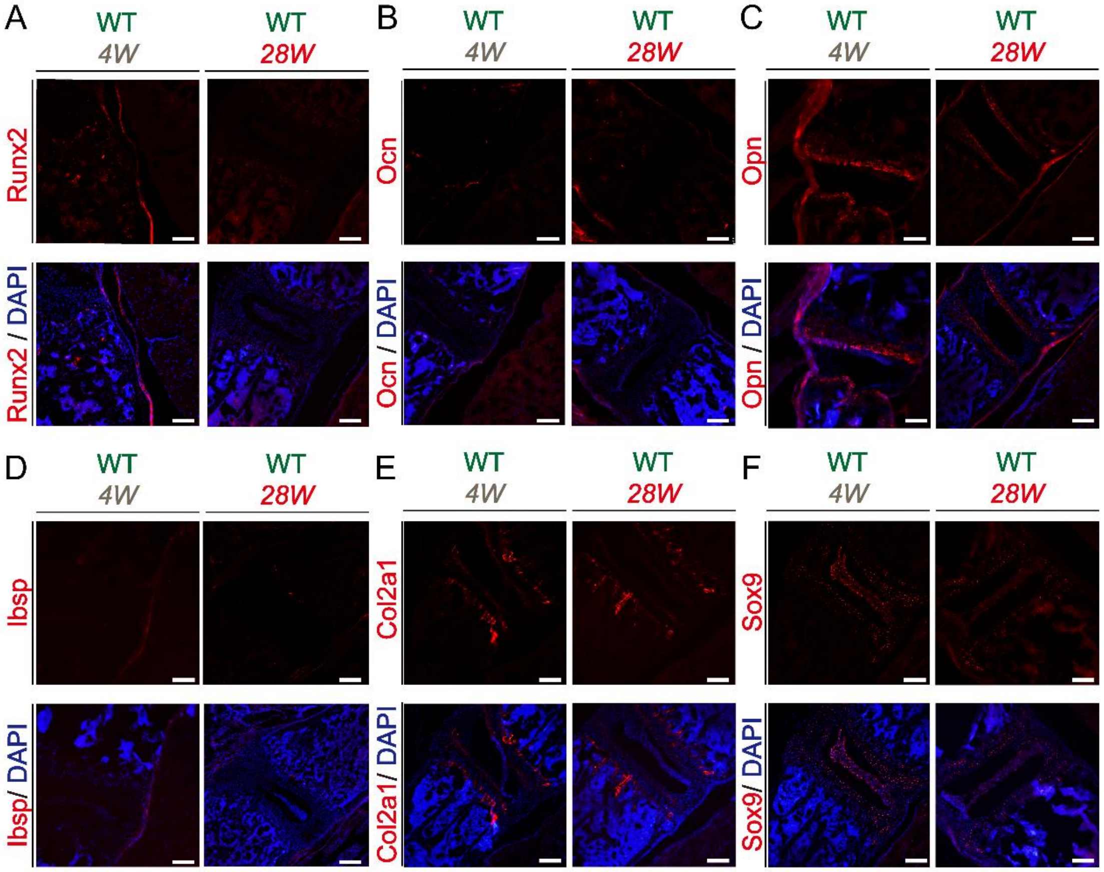


**Supplementary figure 4. Runx2, Ocn, Opn, Ibsp, Col2a1, and Sox9 immunofluorescence of wild-type mice at 4 and 28 weeks for comparison of corresponding time points.** (A-D) Runx2, Ocn, Opn, Ibsp immunofluorescence of wild-type. (E-F) The expression of Col2a1 and Sox9. Scale bars = 125 um, n=6.

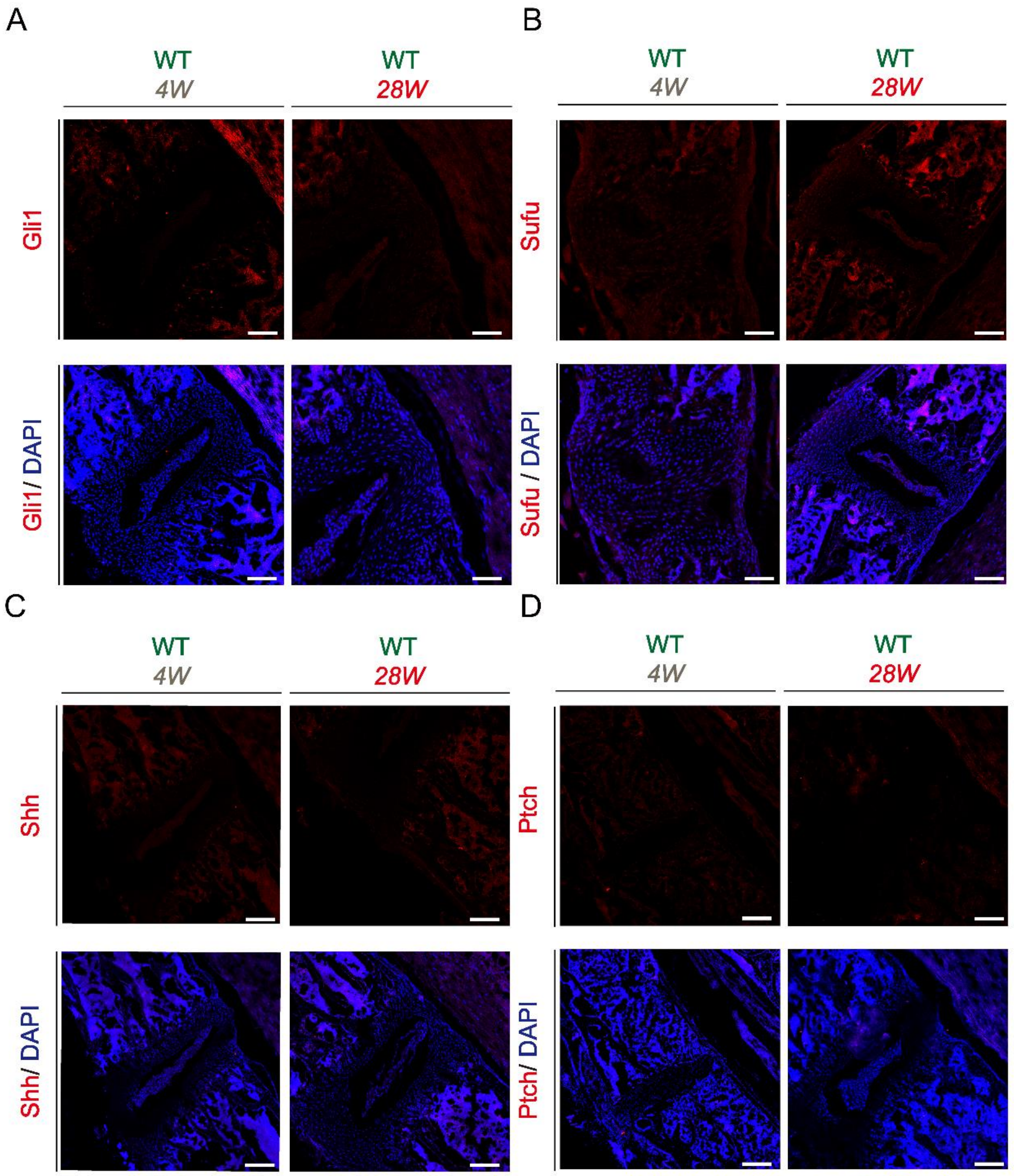


**Supplementary figure 5. Gli1, Sufu, Shh, Ptch immunofluorescence of wild-type mice at 4 and 28 weeks for comparison of corresponding time points.** (A-D) Gli1, Shh, Ptch are barely expressed in WT mice at 4 and 28 weeks, while Sufu is expressed in vertebral bodies and upper and lower endplates at 4 and 28 weeks. Scale bars = 125 um, n=6.

## Supplementary Table: primers used for RT-qPCR.

| | |
|---|---|
| Enpp1 | F:5′-CAGTCAGCAGCGAGGTCATCAA-3′<br>R:5′-CGAGCAGCAGGTCCATACACAA-3′ |
| Col2a1 | F:5′-GTGGAGCAGCAAGAGCAAGGAA-3′<br>R:5′-TCAGTGGACAGTAGACGGAGGAAA-3 |

| | |
|---|---|
| Col10a1 | F:5′-GGATGCCGCTTGTCAGTGCTAA-3′<br>R:5′-GGTCGTAATGCTGCTGCCTATTGTA-3 |
| Acan | F:5′-CCGACATAGACACAGGCACTTCA-3′<br>R:5′-GCTGATGGCAACATTCACCTCTG-3 |
| Sox9 | F:5′-ATCTGAAGAAGGAGAGCGAGGAAG-3′<br>R:5′-TGTTCTTGCTGGAGCCGTTGA-3 |
| Mmp13 | F:5′-CACAGTTGACAGGCTCCGAGAA-3′<br>R:5′-CCACATCAGGCACTCCACATCT-3 |
| Col1a1 | F:5′-ACAGGCGAACAAGGTGACAGAG-3′<br>R:5′-ACCAGGAGAACCAGGAGAACCA-3′ |
| Spp1 | F:5′-TGACGATGATGATGACGATGGAGAC-3′<br>R:5′-TGTAGGGACGATTGGAGTGAAAGTG-3′ |
| Alpl | F:5′-ACATCCCAGAAAGACACCTTGACT-3′<br>R:5′-TTCACCGTCCACCACCTTGTAG-3′ |
| Bglap | F:5′-CAAGCAGGAGGGCAATAAGGTAGTG-3′<br>R:5′-GATGCGTTTGTAGGCGGTCTTCA-3′ |
| Runx2 | F:5′-ACCAGCAGCACTCCATATCTCTACT-3′<br>R:5′-CCGTCAGCGTCAACACCATCAT-3′ |
| Gli1 | F:5′-AAGCCTGAGCCTGAGTCTGTGTA-3′<br>R:5′-CCGAAGGTGCGTCTTGAGGTTT-3′ |
| Ptch1 | F:5′-TGGTTCTGCTGCCTGTCCTCTT-3′<br>R:5′-TGCTGTGCTTCGTATTGCCTGAG-3′ |
| Hhip | F:5′-CTGCTCTTTGGTCCTGATGGCTTT-3′<br>R:5′-GGCTGGTTGGTGCTGTTGAAGT-3′ |
| Shh | F:5′-GCGGCAGATATGAAGGGAAGAT-3′<br>R:5′-GCCACTGGTTCATCACAGAGAT-3′ |
| Sufu | F:5′-ACATCCCTGAGCACTGGCACTA-3′<br>R:5′-CACTTGGTCCGTCTGTTCCTGTAA-3′ |
| Gli2 | F:5′-CTCTCACCTCCATCAGCACCAT-3′<br>R:5′-CCTCAGCCTCAGTCTTGACCTT-3′ |
| Smo | F:5′-TGCCCGCCGAGAGATTGTTT-3′<br>R:5′-GAACCAGACTACTCCAGCCATCA-3′ |
| Pth1h | F:5′-CCGTTTCTTCCTCCACCATCTGATC-3′<br>R:5′-TGCCCTCATCGTCTGACCCAAA-3′ |

| | |
|---|---|
| Gapdh | F:5′-GACTCCACTCACGGCAAATTCAAC-3′<br>F:5′-AGACACCAGTAGACTCCACGACATA-3′ |
| P16 | F:5′-TGAGGGTTTTCGTGGTTCAC-3′<br>R:5′-TGGTCTTCTAGGAAGCGGC-3′ |
| P21 | F:5′-GATGAGTTGGGAGGAGGCAG-3′<br>R:5′-CTGAGAGTCTCCAGGTCCAC-3′ |
| P53 | F:5′-ATGATTTGATGCTGTCCCCG-3′<br>R:5′-CAAGAAGCCCAGACGGAAAC-3′ |

*Table: Primers used for RT-qPCR.*
